# Effectiveness and legitimacy of forest carbon standards in the OTC voluntary carbon market

**DOI:** 10.1186/1750-0680-6-4

**Published:** 2011-08-17

**Authors:** Eduard Merger, Till Pistorius

**Affiliations:** 1 UNIQUE GmbH, Schnewlinstrasse 10, 79098 Freiburg, Germany; 2 Institute of Forest and Environmental Policy, Albert-Ludwigs University of Freiburg, Tennenbacherstr. 4, 79106 Freiburg, Germany

## Abstract

**Background:**

In recent years, the voluntary over-the-counter (OTC) carbon market has reached a significant market volume. It is particularly interesting for forest mitigation projects which are either ineligible in compliance markets or confronted with a plethora of technical and financial hurdles and lacking market demand. As the OTC market is not regulated, voluntary standards have been created to secure the social and environmental integrity of the traded mitigation projects and thus to ensure the quality of the resulting carbon credits. Building on a theoretical efficiency-legitimacy framework, this study aims to identify and analyse the characteristics and indicators that determine the efficiency and organisational legitimacy of standards for afforestation/reforestation carbon projects.

**Results:**

All interviewed market actors consider third-party certification and standards as a crucial component of market functionality, which provide quality assurance mechanisms that reduce information asymmetries and moral hazard between the actors regarding the quality of carbon credits, and thus reduce transaction costs. Despite this development, the recent evolution of many new and differing standards is seen as a major obstacle that renders it difficult for project developers and buyers to select an appropriate standard. According to the interviewed experts the most important legitimating factors of standards are assurance of a sufficient level of quality of carbon credits, scientifically substantiated methodological accounting and independent third-party verification, independence of standard bodies, transparency, wide market acceptance, back-up of the wider community including experts and NGOs, rigorous procedures, and the resemblance to the Afforestation/Reforestation (A/R) CDM due to its international policy endorsements. In addition, standards must provide evidence that projects contribute to a positive social and environmental development, do no harm as a minimum requirement and build a strong track record of successful projects. Project developers require clear, easily and practically applicable standards at lowest possible costs with a high potential in order to achieve good carbon prices, while buyers require that standards are legitimate, credible and that no public criticism arises when carbon credits are purchased from projects certified by a certain standard.

**Conclusions:**

Despite the fragmented and immature state of the OTC market, standards act as 'market-making' intermediaries and contribute to the quality and transparency of the OTC market. However, the variety of different standards imposes new hurdles for their efficiency and often creates confusion instead of confidence among potential buyers. Despite the lacking legitimacy of the standards, pressures from the institutional environment on standards ensure a minimum quality of carbon credits (including positive social and environmental impacts of carbon credits) that serves as an insurance mechanism for the integrity of standards. Its unregulated nature and the pressure from an increasingly competitive environment provides innovative space to deliver efficient certification procedures without imposing unreasonably high transaction costs on market actors. Furthermore, voluntary standards imply a more innovative certification approach, as one legal authority could do, because standards have to compete for adopters backed by civil society organisations. Thereby, the forest sector in OTC voluntary market bears great opportunities to provide the forest sector with crucial lessons for international climate policy and governmental institutions when designing regulation for forest regulation such as international and national REDDplus schemes.

## Background

Increasing evidence of rapid climate change has further fuelled the search for options to mitigate emissions and stresses the need to make use of the potential forests provide in this context [1]. Although the options have been discussed for years, the considerable potential predicted by the IPCC to reduce emissions from deforestation and degradation in developing countries (REDDplus) as well as to enhance carbon sequestration through afforestation/reforestation (A/R) activities or sustainable forest management (SFM) is still far from being realized [2]. While REDDplus still depends on resolving many open questions, the project-based A/R Clean Development Mechanism (CDM) of the Kyoto Protocol has so far only yielded a few projects. Reasons are the scientific complexity associated to quantifying and accounting for the carbon fluxes, the risks associated with non-permanence, costly and lengthy bureaucratic rules and modalities, and most importantly the temporary accounting of Certified Emission Reductions (CERs) and the higher transaction costs compared to other CDM project types. Particularly the latter constraints make such credits unattractive to buyers and CDM investors [2].

Parallel to the launch of several compliance carbon markets, an over-the-counter (OTC) voluntary carbon market has evolved that until today constitutes the largest market for forestry-based carbon credit transactions [3] - one of the reasons being that the largest allowance-based Kyoto cap-and-trade carbon market, the Emission Trading Scheme (ETS) of the European Union, has deferred to include forestry under its framework. Since 2005, Voluntary/Verified Emission Reductions (VER) transactions within the forest and land use sector rose from 3.5 MtCO_2_-eq in 2006 (36% market share) to about 28.4 MtCO_2_-eq in 2010 (46% market share), while the REDD and A/R sectors have been the major sources of VERs with 17.8 and 6.8 MtCO_2_-eq respectively in 2010 [4,5]. The remaining forestry-based transactions resulted from projects aiming at reducing emissions from deforestation or enhancing forest carbon stocks through improved forest management (IFM).

The OTC voluntary carbon market is characterised by fragmentation and diversity of actors with different organisational contexts and is subject to dynamic development. VERs are created on a project basis and carbon credit exchanges occur voluntarily and individually. There is neither a body in charge of regulating carbon credit generation and exchanges, nor do commonly accepted formal rules on the quality and trade of carbon credits exist. In light of its unregulated nature and the growing amount of transactions, public scrutiny and media attention have been increasingly questioning the quality of forest carbon offsets, doubting whether these are real, additional and harmless to people and the environment. Further criticism concerns the complexity of measuring, monitoring and verifying forest carbon stock changes as well as adequately coping with the forestry-specific uncertainties of permanence, leakage and social and environmental integrity of forest carbon projects.

In order to address these issues, different non-governmental organizations have developed certification schemes during the last few years. They aim to set standards for different aspects, such as greenhouse gas (GHG) quantification, for social and environmental aspects, for monitoring, reporting and verification, as well as for registration of VERs. Although these standards today constitute a crucial component of the market's functionality and legitimacy, the variety and diversity of standards with various scopes and approaches has failed to generate transparency and has led to confusion among market participants regarding the assessment of the quality and integrity of the traded credits [6-9].

The paper at hand presents the results of a recent study carried out at the University of Freiburg. Its objective was to identify and analyse the characteristics and indicators determining the efficiency and organisational legitimacy of standards for A/R carbon projects on the basis of a theoretical efficiency-legitimacy framework. The main underlying assumptions were that single or combined application of different forest carbon standards in the voluntary carbon market can only succeed if they

• allow for the generation of carbon credits at low transaction costs and thus facilitate efficient transactions between project developers and buyers,

• ensure the quality of carbon credits (VERs) as well as social and environmental benefits, and

• if they succeed in building a significant level of credibility and market acceptance.

In the following we present the scope of forest carbon standards in the OTC market and elaborate on the theoretical efficiency-legitimacy framework, the methodological approach and the results. Finally, we discuss whether and how forest carbon standards in the voluntary carbon market operate as innovative market makers with regards to their efficiency and organisational legitimacy, followed by conclusions on the role of forest carbon standards in the OTC market.

### Scope of Forest Carbon Standards in the OTC Market

Carbon dioxide is an intangible and invisible commodity. Therefore it is a challenge to determine the quality of carbon credits; both, value and integrity are determined by the elements of how they are "defined, represented and guaranteed" [10]. According to WWF, the quality of a forest carbon offset depends on whether it is "real, additional, measurable, independently verifiable, permanent, unique and [whether it] has sustainable development benefits" [11]. Considering these attributes, there is always the problem that the information regarding the quality of a carbon credit is asymmetrically distributed between the project developers and the buyers.

The production of low quality credits normally costs less than that of high quality credits. If quality does not matter, there is a considerable risk of opportunistic behaviour of carbon credit suppliers as they have a rational interest to achieve high revenues and at the same time to minimize costs. As a result, high quality projects become less competitive in comparison to low quality projects [12]. At the same time, the buyer - also acting rational - will choose the cheapest credits, eventually leading to a "race-to-the-bottom" because he is not aware of the actual quality of a carbon offset. Since the purchase of carbon credits in the OTC market is voluntary, another option for the buyer would be not to purchase any carbon credits at all. In order to fill this quality assurance gap, recently evolving third-party standards with different foci act as regulative market institutions: they aim at ensuring a minimum quality of carbon offsets, seek to provide more transparency and functionality, and try to build confidence among market actors [7,9,13].

### Fundaments of Forest Carbon Standards

Since there is no institutionalized framework for standards in the voluntary market, the development of standards is not subject to any formal regulation. Nonetheless, in order to guarantee harmonised, comparable and high quality carbon credits, certification must ensure that carbon credits meet the above mentioned characteristics in order to legitimise forest carbon offsetting activities. With respect to these quality attributes, Broekhoff distinguishes between three fundamental components of standards that comply with WWF's meta-standard framework and the WWF forest carbon standards assessment guide [10,11,14]; these components are comprised of

(1) carbon accounting (additionality, baseline, leakage, quantification and accounting of GHG benefits, permanence, social and environmental performance)

(2) monitoring, reporting and verification (MRV), including guidance, third-party validation and verifications, and accreditation of validating/verifying institutions

and (3) registration and enforcement (preventing double counting, utilisation of independent registries, guidance on ownership and liabilities of reversal of GHG benefits).

Carbon accounting standards must provide consistent guidance for the monitoring, reporting and verification (MRV) of the actual carbon sequestration or emission reductions in all relevant carbon pools, aiming to ensure that the effects are real, additional, permanent, and measurable. This includes the identification of geographical project boundaries, baseline scenarios, additionality, leakage, and permanence [11]. Standards must further define rules that enable independent third-party auditors to verify the actual emission reductions and carbon removals as outlined in the project design documentation [10]. Finally, as these third-parties should facilitate objective project audit, they must be accredited and provide evidence of sufficient expertise to conduct certifications in accordance with a specific accounting standard. In addition to these accounting issues, the WWF meta-standard framework (MSF) requires "credible and comprehensive standards" to also include criteria, tools and features that guarantee and assess the project's environmental and social integrity, taking into account that forest carbon projects are embedded in large spatial and socio-economic environments.

Although there is no regulatory authority in the OTC market, registration and enforcement is another crucial element in order to cope with the risk that carbon credits are sold or counted more than once. Such "double counting" may also occur if projects are implemented in annex I countries that are subject to Articles 3.3 and 3.4 of the Kyoto Protocol [15], e.g. when carbon credits are registered in both the respective national carbon registry and in addition in a voluntary carbon market registry. The same risk occurs if projects are registered in two different voluntary registries. Moreover, unclear ownership of carbon credits may result in conflicts over the right to sell these.

In practice, the standards presently active in the voluntary market provide different guidance and approaches for dealing with these fundamental issues. Currently, three types of standards integrate the described pivotal components to different degrees. Thereby the differentiation is made between modular pure greenhouse gas (GHG) accounting standards that issue certified VERs, pure social and environmental performance standards, and so-called holistic standards that issue VERs and require that projects generate positive social and environmental impacts (table 1). In order to meet all the requirements for high quality carbon credits, such standards should be comprehensive or can be combined through double certification - as it often occurs in practice, because out of nine forest carbon standards that are currently applicable, there is currently no standard that entails all essential components.

**Table 1:** Overview fundamental components of forest carbon standards

Standard	A/R CDM	American Carbon Registry (ACR)	CarbonFix Standard (CFS)	Climate Action Reserve (CAR)	Climate, Community & Biodiversity Standards (CCBS)	ISO 14064-2/3:2006	Plan Vivo Standards	Social Carbon Standard	Verified Carbon Standard (VCS)
**Eligible project location**	Non-Annex I countries	Globally	Globally	United States of America	Globally	Globally	Developing countries	Globally	Globally
**Baseline/Additionality**	✔	✔	✔	✔	✔	o	✔	o	✔
**Leakage**	✔	✔	✔	✔	✔	o	✔	o	✔
**Quantification and accounting of GHGs**	✔	✔	✔	✔	✓	✔	✔	o	✔
**Permanence**	✔	✔	✔	✔	Not applicable	o	✔	Not applicable	✔
**Environmental and social performance**	✓	✓	✔	✓	✔	o	✔	✔	✓
**Monitoring guidance**	✔	✔	✔	✔	✔	✔	✔	✔	✔
**3^rd^-party validation/verification**	✔	✔	✔	✔	✔	✔	✓	✔	✔
**Accreditation of validators/verifiers**	✔	✓	✓	✓	✓	✔	✓	✓	✔
**Prevention of double counting**	w	✔	✔	Not applicable	Not applicable	Not applicable	✔	Not applicable	✔
**Independent registry**	✔	✓	✔	✓	Not applicable	Not applicable	✔	✔	✔
**Guidance on ownership & liabilities for GHG reversals**	✔	✔	✔	✔	✓	Not applicable	✔	Not applicable	✔

✔ = The standard sets regulation on the fundamental component

✓ = The standard partially sets regulation on the fundamental component

○ = The standards does set regulation on the fundamental component

Not applicable = Due to the scope of the standard, this criteria does require regulation

The table is based on the review of the respective standards documents and the respective websites of each standard [16-28]

### Theoretical Background: An Efficiency-Legitimacy Framework

The "efficiency-legitimacy framework" is based on the New Institutionalism in organisational analysis and the concept of "societal sectors" or "organizational fields" [29,30]. A societal sector is defined by "(1) a collection of organizations operating in the same domain, as identified by the similarity of their services, products or functions, (2) together with those organizations that critically influence the performance of the focal organizations: for example, major suppliers and customers, owners and regulators, funding sources and competitors." Scott and Meyer distinguish between two coexisting societal sectors in which organisations' activities take place, such as a competitive, efficiency-driven and an institutional sector [30]. The competitive efficiency-driven sector is a sphere where a "product or a service is produced and exchanged in a market such that organizations are rewarded for effective and efficient control of their production systems," seeking to produce in a more competitive way than their competitors with the objective to maximise their returns on investment.

In contrast, the institutional environment is "characterised by the elaboration of rules and requirements to which individual organisations must conform if they are to receive support and legitimacy" [30]. Generally, those organisations that adopt processes and structures complying with the rules and requirements of the institutional environment tend to be more successful. The sources of these rules and socially defined categories may originate from different authorities such as the state, trade associations, or generalised belief systems that determine a legitimate organisational structure, activities, and other sources.

In a competitive efficiency-driven and an institutional environment of organisations, the pressure to conform to institutional rules often prohibits efficiency because actors and organisations are subject to a continuously changing institutional environment and have to simultaneously withstand competition from other organisations [31]. They "compete not just for resources and customers, but for political power, and institutional legitimacy, for social as well as economic fitness" [29]. In this light, the underlying rational choice paradigm of bounded rationally builds a further corner stone of the theoretical framework - actors do not have perfect information; they behave "*intentionally *rational, but only *limitedly *so" [32]. This implies that even though they seek to make rational decisions, the lack of time to gather and process information as well as to sufficiently evaluate all possible alternatives, constrains actors to behave entirely rational [33]. Accordingly, the actors are forced to consider market transaction costs in their decision processes, arising from [34]:

• search and information costs for potential trade partners and initiation of contracts,

• costs from closing contracts including negotiations and decision-making,

• costs that appear from monitoring and enforcing contracts, and

• costs of making and sustaining social relationships.

The organisational set-up and governance structures determine the efficiency of transactions and it is assumed that costs associated with opportunism ("self-interest seeking with guile" that may arise from lying, stealing, cheating or distorted disclosure of information) and bounded rationality are the reasons why organisations can economise on transactions [31,35]: organisations "arise and persist when they confer benefits greater than the transaction costs incurred in creating and sustaining them" [36]. According to Richter and Furubotn, in a setting with positive transactions costs and bounded rationality of market actors, a market can be regarded as an organisation *per se *or as a social network of actors who conduct repetitive exchanges [34]. In order to function, there has to be an institutional framework which consists of a comprehensive system of conventions, norms and regulations. Such an institutional framework of a market is developed and established by so-called "market makers": governments, trade associations, producers, brokers, and intermediaries. Therefore, markets are characterised by a coexistence of competition and cooperation which is based on a common and accepted set of market arrangements. Within this institutional framework, the rationally behaving market actors seek to conduct market transactions at the lowest possible transaction costs to maximise their returns on investment [34].

According to Leland, unregulated markets become inefficient when sellers and buyers are asymmetrically informed about the quality of products [37]. Such lack of information and transparency complicates potential market transactions and results in higher costs and also in adverse selection. Suppliers have an incentive to deliver low quality products while buyers, who are incapable of determining the quality of a product, seek to pay as little as possible. Biglaiser attributes the task of ensuring quality to the intermediaries through experts which assess and certify the quality, and thus help reducing the mentioned informational asymmetries [38]. The intermediary has an incentive to deliver high quality as "the intermediary who sells a low-quality product suffers a loss of reputation, thus loses customers for all other products" [38].

This theoretic approach does not yet consider "moral hazard" - a concept originating from contract theory, which refers to the problem that after the finalisation of a contract between an agent (supplier) and a principal (buyer), the agent has more information and therefore the possibility to act opportunistically, e.g., by cheating or disclosing false information [34]. It may require significant and costly efforts to observe and monitor the characteristics of trading partners and to monitor the enforcement of contracts. Addressing moral hazard and opportunism, Spulber characterises intermediary firms as "market-commitment devices" that reduce transaction costs and contractual opportunism [38]. The obviation of moral hazard and opportunistic behaviour by intermediaries is assured through the design, control and monitoring of appropriate contracts that comprise binding contractual commitments between buyers and sellers. Thereby, intermediaries seek to build a good reputation and credibility as they conduct larger volumes of transactions on which they economise.

Since organisations are embedded into institutional environments, they must not only adapt on the existent institutional environment, but also should "modify their environment in order to maintain competitive advantages" [39]. To persist in the market, organisations seek legitimacy to avoid sanctions from the legal, social and economic actors [40]. Suchman defines organisational legitimacy as "a generalized perception or assumption that the actions of an entity are desirable, proper, or appropriate within some socially constructed system of norms, values, beliefs, and definitions" [41]. Three essential components determine organisational legitimacy: "the characteristics of the institutional environment, the organization's characteristics and actions, and the legitimating process by which the environment builds its perceptions of the organization" [42]. With respect to the characteristics of the institutional environment, organisations are influenced by various actors and are backed by various resource providers. In order to receive legitimacy and support by the immediate audience and the wider political, economic and social community of the organisation, they must

• incorporate legitimizing elements complying with formal and collectively accepted structures of the institutional environment,

• adopt external assessment criteria that define the value of structural elements or external constituents of the organisations policy-making structures,

• engage with other institutions, e.g. centralised states, trade unions or associations [41].

In a nutshell, to establish a moral legitimacy organisations must prove the usefulness of their operations, outputs and goals though a positive normative third party evaluation. Moral legitimacy can be considered as "sociotropic": internal participants and external constituents make judgements in accordance with their socially constructed value systems assessing organisations' societal benefits [41].

## Methods

The outcomes of this explorative study result from a qualitative research design based on various sources [43-45]. After a thorough literature review, 13 structured expert interviews were carried out in 2010 via telephone with actors actively engaged in the forest sector of the OTC market. In the selection process, market actors were approached with a proven personal track record in the forest carbon market sector, e.g. by developing or purchasing forest carbon credits, applying the standards or by publishing research on this topic. After that, three major groups were selected that represent the competitive and institutional environment of the voluntary carbon market. Among these three groups four project developers of A/R carbon projects, four buyers of forest carbon credits and five other market stakeholders were interviewed. Although the latter group, labelled "quality guarantors for civil society", is not directly involved in the production and purchase of forest carbon credits, it is crucial in the context of the topic because it scrutinises, evaluates, and communicates the quality and robustness of forest carbon credits, projects and standards. This group of interviewees consists of an environmental NGO, a scientist, a media representative, an independent third-party auditor and a standard setter.

The questions followed a structured interview guide and encouraged the interviewees to elaborate in detail on their personal perceptions and experiences. Subsequently, the transcribed and paraphrased interviews were analysed by conducting a qualitative content analysis which was guided by the aim to embed the results into the developed theoretical efficiency-legitimacy framework and explain the role of forest carbon standards with regard to the research objectives. The main objective was the identification of representative and common perceptions in order to identify and develop interpretative patterns [46]. Thus, the thematic comparison was conducted through the compaction and structuring of the interviews into thematic categories, using MAXQDA, a qualitative data analysis software package for structuring, extracting and analysing information [47].

## Results

This chapter presents the key results of the conducted interviews. Initially, the perceptions of the experts on the A/R sector in the OTC market are presented. Subsequently, the role of forest carbon standards as market regulators is assessed, followed by aspects related to their efficiency and organisational legitimacy.

### A/R carbon sector in the OTC voluntary carbon market

All interviewed actor groups consistently regard A/R and the entire forest sector in the OTC voluntary carbon market as immature, but differentiated between the pre-compliance market in the US and the pure voluntary market: Most research participants consider the US pre-compliance demand as a significant driver of supply in the OTC market. With the expectation of a future compliance regime, it is believed that the forestry sector will play an important role due to its potential to deliver quick and low-cost carbon credits, as well as the generally positive attitude of US buyers towards forestry. However, in 2010 the pre-compliance demand from the US plummeted because the prospects for US federal climate change legislation worsened while simultaneously uncertainties regarding a future US carbon market increased [48]. Concerning the voluntary market, the interviewees indicated that in recent years there has been a lacking supply of high quality third-party certified forest-based credits. They pointed out that during the last three years third-party certification in the OTC market has become a prerequisite to enter the market. They also stressed the lengthy and difficult process from the project concept up to its implementation and that third-party certification can take up to several years. The experts identified four major constraints of the yet immature A/R forest carbon sector: political constraints, technical complexity, high development and implementation costs, and lacking transparency on quality assurance in the OTC market.

An often mentioned political constraint is the ineligibility of forest carbon credits in the EU ETS compliance market despite a perceived increasing recognition that forestry needs to play a key role in climate change mitigation - particularly among European VER-buyers, who historically had a rather negative perception towards forestry for carbon sequestration. The major technical constraints result from the MRV of climate benefits, the unique socio-economic and environmental conditions of A/R projects, the required level of expertise, and the high costs of developing, certifying and implementing projects. The project-specific socio-economic and environmental conditions require individual, long-term designs, considering the long lifetimes of forest projects, as explained by project developers and quality guarantors. Furthermore, in most cases only large-scale projects tend to be financially attractive, thus a certain size of projects needs to be achieved in order to make these feasible. Lastly, the experts from the quality guarantor group and the buyers' group mentioned the lack of robust quality assurance mechanisms and a supply of certified high-quality credits that was not able to meet the demand.

Since all these aspects have to be dealt with, it is a lengthy procedure to prepare appropriate project documentation and to certify A/R projects, impairing their competitiveness compared to other non-forest project types. Consequently, only large-scale A/R projects tend to be attractive for investors, as these are capable of creating the significant upfront finance for covering the arising costs for project certification and the subsequent MRV. In addition, the long duration of forest projects delay the revenues from such projects, remarkably constraining investments in such projects.

### Forest Carbon Standards in the OTC Market

After identifying the constraining aspects of the A/R sector, the focus was to investigate actors' perceptions towards existing forest carbon standards with respect to their role as market regulating entities and their competitive forces. In addition, the views on organisational legitimacy were assessed.

All interviewed market actors regard certification and standards as a crucial component of market functionality, which provide a quality assurance mechanism that reduces information asymmetries between the actors regarding the quality of carbon credits. Particularly for brokers, retailers and wholesalers, standards were regarded as a fundamental underpinning to purchase and subsequently sell the credits. For project developers, standards provide a guiding framework for the minimum quality of projects that ought to be met in order to successfully enter the market and to sell the credits. The standards also provide guidance for principles and procedures that legitimise the project activities to other stakeholders of the projects. According to the interviewees, the recent development of different standards in the OTC market has been crucial: credible and widely accepted certification has become a key prerequisite for selling forest carbon credits.

Despite this, the recent evolution of many new and differing standard schemes is considered a major obstacle, creating difficulties for project developers and buyers to select appropriate standards. The availability of the various options comprises uncertainties about the efficacy and credibility of the standard schemes to the market participants. As only few A/R projects have gone through the entire certification and verification procedures yet, only few credits were delivered to the market and accordingly the amount of experiences is rather small. This dynamic market development is regarded by the interviewees as an indicator that market actors have not yet identified a standardised solution; due to this shortcoming, most standards are used and accepted by the majority of the market actors, leading to competition between the standard schemes.

It appears that despite the different standards, there is little confidence that their application provides an effective mechanism for market actors to ensure the quality of projects and forest offsets to the extent that buyers can solely rely on them as quality assurance mechanisms. Project developers and other interviewees indicated that this may be related to the fact that buyers often are not familiar enough with the standards. As a consequence, many buyers undertake additional project analysis and due diligences that increase transaction costs significantly.

### Efficiency of Forest Carbon Standards

Project developers and the quality assurance actors regarded the high quality benchmark set by the A/R CDM for voluntary markets as a major constraint, as it is considered a complex, costly and overly bureaucratic mechanism. The interviewees pointed out that this neither effective nor efficient benchmark unnecessarily complicates the design of voluntary forest standards.

As all land-based projects are embedded in complex socio-economic and large spatial dimensions, such projects must address social and environmental issues in order to reduce the risk associated with the acceptance and involvement of local stakeholders, permanence and leakage. As there exists no accepted "one-size-fits-all"-standard, it is regarded as a further significant and costly impediment that project developers often must use at least two forest carbon standards in order to separately account for carbon benefits and the crucial socio-economic and environmental benefits (table 1). Moreover, the project developers emphasised that the data collection, compilation and analysis require significant labour and time resources, which are the main drivers for the high transaction costs in forestry projects. Consequently, they seek for standards following a comprehensive approach, i.e. a consideration of carbon benefits as well as the social and environmental impacts. To them the application of two different standards requires significant additional work for project documentation and certification that increases transaction costs. However, they also pointed out that these costs also depend on the expertise of the project developers, because low quality project design documents significantly increase the costs of third-party validation and verification.

Since most forest carbon projects and certification activities have begun in the last few years, many project developers are still at the beginning of the learning curve; with increasing experience in the application of standards, they will be able to streamline and optimise methodological and procedural issues and conduct project documentation and certification in a more cost-effective and efficient manner. Regarding the technical constraints of A/R project certification, well experienced actors emphasised the lack of widely applicable and easily available technical and scientific data that are required to make project documentation more complete.

### Organisational Legitimacy of Forest Carbon Standards

Since standards act as quality assurance mechanisms in the OTC market and the buyers so far undertake climate change mitigating activities on a voluntary basis, they must define effective safeguards for the quality of VERs in order to build confidence between buyers and project developers, and also to facilitate market transactions. In this context, the interviewees regarded a certain level of credibility and market acceptance as crucial. However, the perceptions differed on how the standards can achieve an overall credibility and market acceptance.

According to the interviewees, the most important legitimating factors of standards are assurance of a sufficient level of quality of carbon credits, scientifically substantiated methodological accounting and independent third-party verification. In addition, standards must provide evidence that projects contribute to a positive social and environmental development, and do no harm as a minimum requirement. A further legitimating aspect is the organisational background of a standard, in particular the expertise and the backing of reputable entities incorporated in the standard. To meet these criteria standards have to be independent, free of conflict of interests and underpinned by scientific expertise; furthermore they need the recognition of reputable environmental and social NGOs, conveying legitimacy to the standards according to the buyers and quality guarantors. Thus, it can be ensured that interests of the civil society are sufficiently represented and that transparent and rigorous procedures and principles of standard schemes are implemented.

Moreover, buyers demanded that standards need to transparently demonstrate their long-term success and applicability by building a track record of successful projects and carbon transactions that deliver permanent climate, social and environmental benefits. This long-term viability of projects under a certain forest carbon standard is regarded as a decisive factor and plays an important role in the decision-making to select standards for the certification of A/R projects in the OTC market. Additional significant legitimating power originates from the actual consumers who decide on standards' integrity by purchasing credits from projects that are certified by specific standards. Last but not least, a critical aspect of legitimacy is the governmental endorsement of standards mentioned by buyers and quality guarantors. The governmental support or acceptance could considerably increase the integrity of standards, their market acceptance and confidence. This is particularly crucial to actors with pre-compliance pursuits. Therefore, standard developers should also undertake efforts to become acceptable and suitable for public activities and governmental authorities.

## Discussion

The results show that the OTC market is still in an immature state, despite the development of standards and registries that have been an essential advancement of the market's integrity. The variety of standards undermines the credibility of the entire forest carbon sector and poses barriers to market actors to enter the market and to operate efficiently. In the future, having the choice to use different but complementary standards may yield potential advantages, but currently their multitude is perceived as a constraint. Another impediment is the fragmentation of the market, as there are no powerful entities that influence and drive the development of widely accepted standards that lead the market's development and engage in the legitimating process of standards by developing and promoting these. In the following, we discuss forest carbon standards' role as market makers, their efficiency and their organisational legitimacy based on the theoretical efficiency-legitimacy framework and the results from the interviews.

### Standards as market makers

In view of the presented theoretical efficiency-legitimacy framework, standard schemes act as market making intermediary organisations that seek to guarantee homogenous and transparent quality of forest carbon credits by facilitating "coordination and cooperation on a global scale" [49]. In practice, standards seek to regulate the functionality of the market by facilitating transactions of carbon credits and reducing the required efforts in order to obtain information on the potential trading partners and the quality of carbon credits. This includes less asymmetric information and reduced moral hazard between buyers and project developers of forest carbon offsets which reduces transaction costs. The voluntary nature of standards implies that they are not enforced by any legitimate authorities, but actively demanded by private profit and non-profit organisations alike. Generally, standardisation is a mean of facilitating market exchanges if there is no "legal centre of authority" and "provides coordination and control on the basis of agreed procedures," quality and definitions [50,51]. The impetus to create standards is normally higher if the market is fragmented, not transparent and comprised of various actors or organisations that do not have the power to drive and shape the market. Standardisation can lead to more uniform quality of products and rules, and eases the confidence of buyers and project developers on the integrity of the market [52].

In general, the better a standard is known, the larger the amount of adopters will be if it produces similarities among them [52]. In contrast, if there are many competing standards, there is the likelihood of diverse quality and a varying degree of uniformity. Uniformity will increase if certain standards gain more adopters and if standards have more followers. Thus, it is likely that new market actors will also adopt these standards that give way to monopoly of them, reducing the pressure to be innovative and to be efficient [52]. This implies trade-offs between competition and innovation of standards. On the one hand, strong competition leads to diverse quality of forest carbon credits that results in a non-standardised product imposing high transaction costs on market actors to evaluate the quality and navigate among the standards. On the other hand, competition is a pivotal driver of innovation as standards in the OTC market compete for potential adopters seeking to develop the most attractive solutions to make carbon credits fungible in the market.

### Efficiency

According to the theory of New Institutional Economics, efficient standardisation relates to the ability of standards to facilitate exchanges between buyers and project developers without imposing too high transaction costs [33]. This corresponds to low costs for standards' application, avoidance of moral hazard and removal of informational asymmetries. Therefore, one major aspect of efficiency seeking forest carbon standards is the reduction of costs for project certification and the reduction of information asymmetries on the quality of a carbon credits between the project developers and the buyers. Efficient standards create confidence among the actors, help to attract buyers, and ensure quality. The results of this study show that the current forest carbon standards are not yet able to build sufficient confidence among potential buyers as they do not solely trust certification to the standard schemes and undertake own additional efforts to assess the quality of projects. Reasons are the variety, low transparency and the incomplete nature of the many standards (table 1). Often this leads to the utilisation of at least two standards schemes for A/R projects to ensure carbon accounting on the one hand, and social and environmental integrity on the other. This leads to higher transaction costs through the duplication of work associated with preparing project documentation and certification procedures. The additional transaction costs are passed on to buyers, which further contributes to the inefficiency of the A/R sector.

The results of this study also indicate a prevailing lack of confidence of market actors regarding the integrity and efficacy of the current active standards due to their relatively recent establishment and few experiences with forest projects that have lifetimes and crediting periods of several decades. The standards have not had the chance yet to prove their ability to guarantee and enforce that A/R projects are maintained in the long-term and that the sequestered carbon is stored permanently. There appears to be the need to integrate the pivotal components of standards such as efficient and effective guidance and procedures on carbon accounting (MRV), social and environmental aspects, as well as registration and enforcement in order to provide market actors comprehensive and efficient standards. Considering the existing market infrastructure, this could be achieved through consolidation and reconciliation of standards' requirements and procedures, eventually leading towards quality harmonisation of the supplied A/R credits to the market. Further, standards need to be linked to independent registries that help to coordinate the supply and demand; this would help to reduce the current market transaction costs and increase market transparency.

Despite the importance of more harmonised standards, competition between standards must be regarded as a crucial component of innovation that drives efficient market development and functionality through the requirement of standards to compete for adopters in a constrained rationally behaving environment seeking for the highest possible gains by applying the standards. Otherwise, there is the risk that "once a standard has become established and accepted, it is very difficult for new and better ways to win market approval. Thus, standardisation may not only promote but also inhibit competition and innovation" and trade-offs between competition and innovation appear in the OTC market [52].

### Organisational Legitimacy

Approaching the non-competitive institutional environment, standards have to prove that they are "desirable, proper, or appropriate" within the socially constructed "norms, values, beliefs and definitions" [41]. As the application of forest carbon standards is neither induced nor accompanied by any mandatory requirement, legitimacy may be claimed by developing organisations for their standard by seeking to "reflect what is best or at least desirable, or that they are objectively appropriate" [50]. The voluntary character implies an apparently less complicated way to establish a certain degree of organisational legitimacy (compared to the complex and lengthy processes of political or mandatory authorities, as there is no sanction for non-compliance with the standards or not applying these. Therefore, actors will disclaim using standards, if these are not beneficial to them [53]. If standards are not useful or accepted, the organisations are less legitimate, and if standards seek to receive acceptance, these must prove that they are "morally right, beneficial for the users, and the like" [50]. In addition, legitimacy originates from the endorsement of forest owners, their strength and costs of a standard [53].

In practice, forest carbon standards are most likely to achieve legitimacy if they are able to guarantee the general quality indicators of a forest carbon offset and through the integration of the described pivotal standards' components. However, in practice the quality of carbon credits is difficult to assess due to the invisible and intangible character of carbon credits, even though A/R projects are certified to a certain standard. Therefore, actors active in the OTC market tend to have evaluation criteria of legitimate standards such as "independence of standard bodies, transparency, wide market acceptance, being understandable, back-up of the wider community including experts and NGOs, rigorous procedures, or the resemblance to the A/R CDM" due to its international policy endorsements.

The usefulness of standards' activities is determined by a range of actors and has differing influence on the legitimacy of standards schemes. Firstly, a great level of legitimating power is given to standards if they incorporate reputable leaders in the management and design of standards, as Brunsson states: "Leaders of standards must enjoy a high degree of legitimacy", because they have the rights to give orders and directives [50]. Another crucial aspect of legitimacy is the involvement of experts and scientific representatives in the standard setting process and their involvement in the management of standards [51]. In addition, Meyer points out the importance of academic knowledge and the development industry that follow the wake of academic science [51].

On the one hand, different actors have different demands on standards with different degrees of legitimating power. Therefore, interactive social and economic processes between the various actors such as experts, academics, project developers, buyers, NGOs, and media determine the organisational legitimacy of forest carbon standards. Project developers require clear, easily and practically applicable standards at lowest possible costs with a high potential in order to achieve good carbon prices, while buyers require that standards are legitimate, credible and that no public criticism is likely to occur from purchasing carbon credits from projects certified by a certain standard. The civil society actors act as quality guarantors and communication tools determining standards' integrity and the usefulness of their activities. Therefore, legitimacy can be increased by the support and involvement of a variety of actors and requires support and endorsement from large corporate groups (powerful agents such as reputable NGOs or governments), as most standards do not have the resources, the authority and power to change others' activities [49]. Particularly governments have important power in signalling that standards are "legitimate and credible governance systems on which private procurers and other buyers can also rely" [53,54].

The involvement of all the different actors in the standard setting processes and their support and moral approval comes at a cost due to the coordinating efforts of different interest groups, the requirement to incorporate these, and the need to communicate and promote standards' usefulness. Thus, legitimacy can be regarded as a significant economic constraint in a competitive environment that requires standards to invest: not for the sake of efficient procedures, but to motivate and attract potential users to apply the standards and receive endorsement by powerful organisations through the incorporation of elements that comply with norms, values and definitions of a widely accepted social system. Particularly in the weak institutional set-up of the voluntary market and the very dynamic nature of the forest carbon sector, the pursuit for legitimacy is a remarkable component of standards' activities. Considering that most forest carbon standards are younger than five years, those that define standards must convince actors to apply them by showing and communicating their effectiveness and their legitimacy. Otherwise, they will not sustain themselves in the market, as they will be regarded as useless.

Specifically in forestry, the standards will most likely require much more time to achieve an adequate level of legitimacy and demonstrate their usefulness due to the long-term horizons of forestry projects. The standards will have to prove that they are able to ensure the long-term sustainability of projects that generate climate benefits as well as social and environmental benefits over decades. This will only be achieved if projects have undertaken more than one third-party verification proving the long-term compliance to the standards and enforcement of the expected climate, social and environmental benefits. Thus, standards must build a track record of projects that generate long-term benefits for the environment and the people delivering the market actors confidence on their legitimacy and efficacy. This will be a long-term interactive process among market actors, civil society organisations, standard setters, academics, and experts who will shape the overall cognitive perceptions on the standards determining their organisational legitimacy.

## Conclusion

The study has shown that OTC voluntary carbon market is still at a dynamic developing stage characterised by the variety of standards, their lacking efficiency and legitimacy - despite the development of certification schemes in recent years. Its unregulated nature and the pressure from an increasingly competitive environment provides innovative space to deliver efficient certification procedures without imposing unreasonably high transaction costs on market actors, and to reduce informational asymmetries and moral hazard. The pressure resulting from the institutional environment on standards to ensure a minimum quality of carbon credits (including positive social and environmental impacts of carbon credits) serves as an insurance mechanism for the integrity of standards. Increased efficiency and legitimacy of the OTC market and a more streamlined quality of A/R carbon credits will further depend on maturation of the forest sector. It remains also to be seen whether the standards will be able to provide more harmonised and reconciled guidance and procedures for A/R carbon project certification, resulting in a more homogenous and complete regulation that is likely to attract new market participants. The voluntary nature implies a more innovative certification approach, as one legal authority could do, because standards have to compete for adopters backed by civil society organisations. Thereby, the forest sector in OTC market bears great opportunities to provide the forest sector with crucial lessons for further development. This innovative and competitive environment of the OTC market serves as a crucial learning ground for international climate policy and governmental institutions when designing regulation for forest regulation such as international and national REDDplus schemes, e.g. through the facilitation of new carbon accounting methodologies, the generation of the first third-party verified REDD carbon credits, and the ensuring social and environmental safeguards for sub-national REDD projects.

## Competing interests

The authors declare that they have no competing interests.

## Authors' contributions

This article comprises a research project carried out by EM and supervised by TP. It presents the main findings that EM has achieved through the interviews carried out for his diploma thesis. All authors read and approved the final manuscript.
